# Surgical Treatment for Non-union of the Great Toe Proximal Phalanx Stress Fracture in an Adolescent Jumping Athlete

**DOI:** 10.7759/cureus.53424

**Published:** 2024-02-01

**Authors:** Yuzuru Sakakibara, Takashi Ochiai, Akira Ono, Akimitsu Oyama, Atsushi Teramoto

**Affiliations:** 1 Department of Orthopedic Surgery, Sapporo Medical University, School of Medicine, Sapporo, JPN; 2 Department of Orthopedic Surgery, Muroran City General Hospital, Muroran, JPN

**Keywords:** teenage athlete, teenager sports injury, great toe, proximal phalanx fracture, non-union

## Abstract

Stress fractures of the proximal phalanx of the great toe are primarily attributed to repetitive shear forces, with the vertical ground reaction forces exerting several times the body weight. In the initial stages of injury, conservative management anticipates bone healing within approximately five weeks, followed by a gradual return to sports activities over an additional five weeks. Athletes presenting with pain in this region warrant a thorough evaluation for stress fractures to initiate timely conservative care. In instances of delayed healing or non-union, surgical intervention is indicated. However, literature on the management and optimal timing of surgery, particularly in adolescent athletes, remains sparse. This case report, complemented by a literature review, offers insights into management based on the patient's clinical course.

## Introduction

Track and field events impose high demands on the musculoskeletal system of adolescent athletes, who are especially prone to sports-related injuries. Among these, the non-union of the great toe proximal phalanx stress fracture is a condition that can significantly impact an athlete's performance due to the toe's critical role in balance and propulsion. Despite this, the condition is relatively rare, with an incidence rate suggested by being notably low when considering the broad population of athletes [1]. This fracture commonly occurs in adolescent athletes, but in most cases, the stress fracture may be missed because of the lack of specific trauma other than continued sports and the mild pain during activity [2]. This case report aims to show a detailed account of the surgical treatment for non-union of the great toe proximal phalanx stress fracture in an adolescent track and field jumper. Our case focuses on a young athlete at the peak of their competitive timing, so it covers the diagnostic challenges, surgical interventions, and rehabilitation strategies employed, along with a follow-up that offers a rare glimpse into the athlete’s return to competition.

## Case presentation

**Figure 1:**
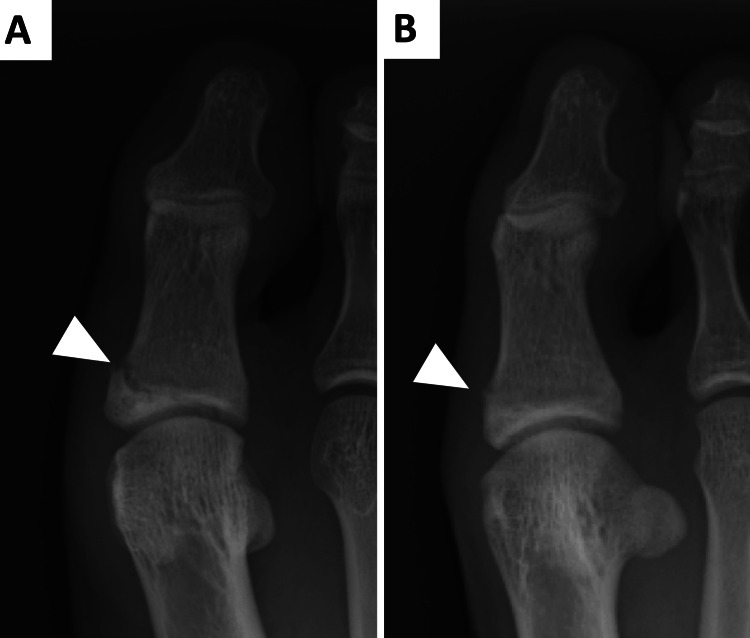
An X-ray at the time of the initial examination showed a crack at the medial base of the proximal phalanx of the great toe A: anteroposterior (AP) view; B: oblique view

A 16-year-old male long jumper on a high school track team with no specific past medical history reported right great toe pain during a sprint dash. Despite the discomfort, he continued his athletic activities without seeking immediate medical attention. One month after the onset of pain, persistent symptoms prompted medical consultation, leading to an initial diagnosis of a fracture in the right proximal phalanx of the great toe via radiography (Figure 1).

**Figure 2:**
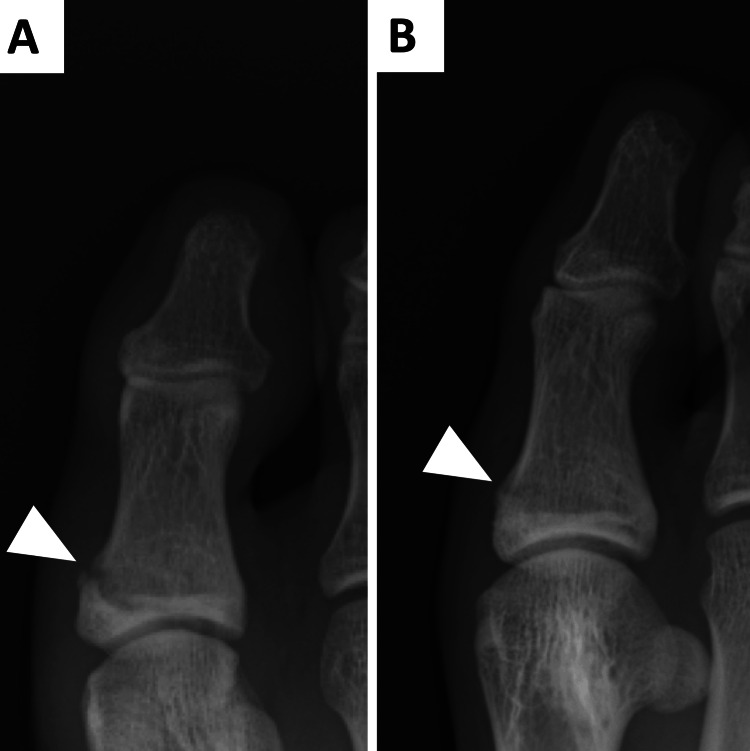
An X-ray six months after the initial diagnosis showed a non-union of the proximal phalanx of the great toe A: anteroposterior (AP) view; B: oblique view

Followed by conservative treatment. Three months post-injury, radiographic evidence of callus formation and symptomatic improvement marked the end of treatment. However, two months later, symptom recurrence necessitated a re-evaluation, revealing a non-union at the initial fracture site, and a radiograph showed the non-union of the proximal phalanx of the great toe (Figure 2).

**Figure 3:**
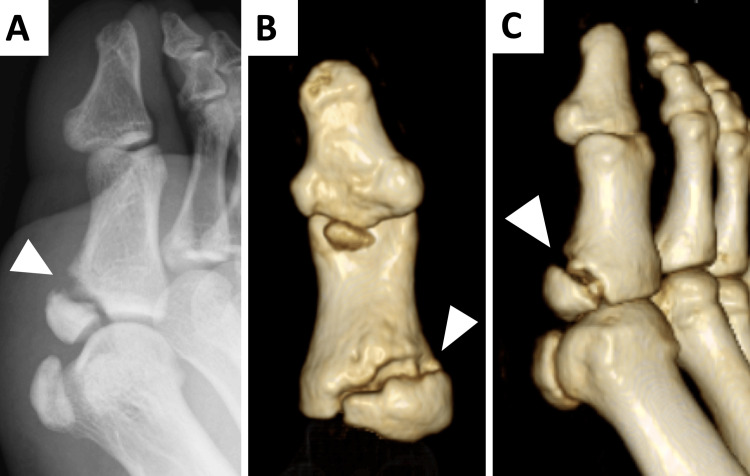
Radiography and CT showed the dislocation of the non-union area of the proximal phalanx of the great toe A: oblique view on X-ray; B: plantar side of the great toe on 3D CT; C: medial side of the great toe on 3D CT 3D: three dimensional, CT: computed tomography

The clinic physician, who had been treating the patient from the beginning, referred the patient to our orthopedic department at this stage. A physical examination in our department identified tenderness at the base of the right great toe. Six months after the injury, with high school qualifiers imminent, conservative management with low-intensity pulsed ultrasound was employed to enable continued sports participation, deferring surgical intervention until after the competitive season. Four weeks after the initial consultation in our department, the patient presented with pain in the same region during a competitive jump, and subsequent imaging demonstrated a dislocation of the non-union site with associated subcutaneous hematoma, necessitating internal fixation surgery. A radiograph showed the dislocation of the medial base of the proximal phalanx of the great toe. CT also showed dislocation of the fracture site in the non-union area at the previous visit (Figure 3).

**Figure 4:**
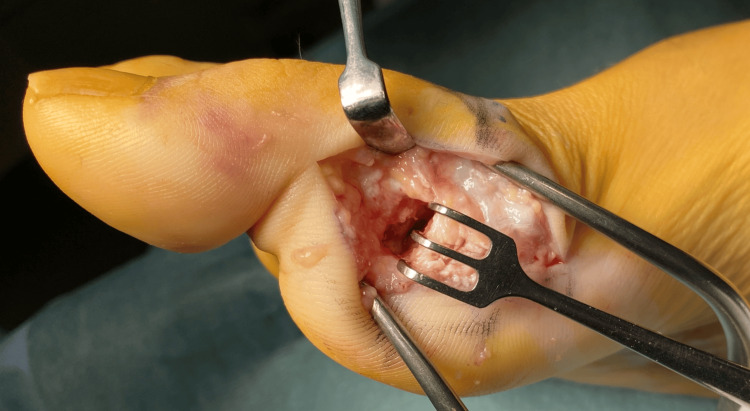
The non-union site was opened, and the fibrotic scar tissue was sufficiently refreshed

At this stage, six months had passed since the initial diagnosis, and since the bone fragments were translating, we considered this case to be non-union and needed surgical treatment. At the time of surgery, the fracture site of the proximal phalanx showed fibrous scarring. After freshening the scarred area, the fracture was fixed using an Acutrak mini screw (Acumed, Hillsboro, OR, USA) and a cannulated screw (Stryker, Kalamazoo, MI, USA) (Figure 4).

**Figure 5:**
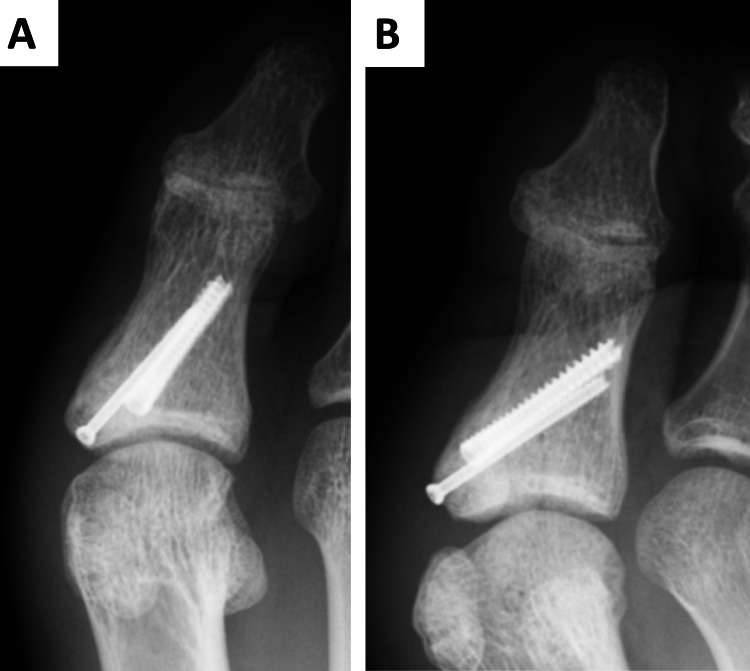
Radiography five months after the operation showed a solid union A: anteroposterior (AP) view; B: oblique view

Postoperative treatment included forefoot loading at four weeks postoperatively, jogging at eight weeks, and returning to competition without restrictions at 12 weeks. Five months postoperatively, X-rays showed solid bony fusion (Figure 5).

The patient returned fully to competition, scoring a perfect 100 on the Japanese Society for Surgery of the Foot scale. At that time, his long jump record was 6 m and 33cm. His pre-injury record was 5 m and 76 cm, which may be a case of improved sports performance due to improved pain from surgical treatment and early rehabilitation intervention.

## Discussion

Stress fractures of the proximal phalanx of the great toe are primarily attributed to repetitive shear forces, with the vertical ground reaction forces exerting several times the body weight [2-4]. Traction and shearing forces of the medial collateral ligament and abductor hallucis tendon on the medial side of the proximal phalanx base are triggers for avulsion fractures of the medial proximal basal phalanx [5-6]. Previous reports have noted that the hallux valgus is associated with stress fractures of the proximal phalanx of the great toe. Some cases require primary surgery for hallux valgus to correct the stresses and shear forces [1,4]. Reports of stress fractures of the proximal phalanx of the great toe are most frequently seen in track and field athletes, with other reports in basketball, kendo, and soccer [1,7-8]. In the initial stages of injury, conservative management anticipates bone healing within approximately five weeks, followed by a gradual return to sports activities over an additional five weeks [2]. Athletes presenting with pain in this region warrant a thorough evaluation for stress fractures to initiate timely conservative care. Most stress fractures of the proximal phalanx of the great toe have been reported to have excellent outcomes with conservative treatment. However, surgical treatment is recommended in cases of delayed diagnosis and treatment or cases involving dislocation. Even with surgical treatment, the return-to-play rate is 100% [1-2,4,7-9]. In the present case, the patient could return to sports at the same level as before the injury. In instances of delayed healing or non-union, surgical intervention is indicated. However, literature on the management and optimal timing of surgery, particularly in adolescent athletes, remains sparse. What is known about this injury revolves around its diagnostic and treatment protocols. Imaging techniques such as MRI and CT scans play a pivotal role in the accurate diagnosis of stress fractures and non-unions of the foot [10-12]. The importance of this study is underscored by its potential to inform future treatments and improve outcomes for young athletes facing similar challenges. By focusing on an adolescent patient, this study is a valuable addition to the field of sports medicine because few case reports focus on this process of returning to target competitions. Additionally, the relationship between stress fractures of the great toe proximal phalanx and concurrent conditions like hallux valgus, as discussed [2], requires further exploration in the context of the younger athletic population. Conservative treatment is effective in many cases, and a four- to six-week non-loading period followed by a four- to six-week staged return to competition is recommended. The indications for surgery are when the athlete wishes to return to play earlier when conservative treatment is ineffective, and in cases of non-union. Since the time required for diagnosis and treatment is eight to 12 weeks [9], it is essential to share this information with the patient and select a treatment that meets the patient's goals for the competition. Whether bone grafting is needed or not is controversial. We didn't perform bone grafting to avoid pain remnants in the recipient site because the patient is a young, long jumper [13-14]. Opening and refreshing the non-union area is essential to obtaining fusion before fixation. This study highlights the importance of knowledge in the diagnosis and surgical treatment of non-union fractures in adolescent athletes, taking into account their unique healing and timing to return to sport and ensure a competitive level. The present findings have the potential to address the unique needs of adolescent athletes, improve outcomes, and shorten the time to return to competition. This report adds to the existing literature and underscores the need for continued research on optimal care for adolescent athletes.

## Conclusions

Stress fractures of the proximal phalanx of the great toe are rare, reportedly accounting for 0.5% of all stress fractures in athletes. However, it is involved in the stepping motion and significantly impacts sports performance, so making treatment decisions is vital. Surgical treatment for non-union of the proximal phalanx of the great toe can ensure bone fusion and an early return to competition when conservative treatment is ineffective in jumping athletes.
